# A data-driven classification of 3D foot types by archetypal shapes based on landmarks

**DOI:** 10.1371/journal.pone.0228016

**Published:** 2020-01-30

**Authors:** Aleix Alcacer, Irene Epifanio, M. Victoria Ibáñez, Amelia Simó, Alfredo Ballester

**Affiliations:** 1 Departament de Matemàtiques, Universitat Jaume I, Castelló, Spain; 2 Institut de Matemàtiques i Aplicacions de Castelló, Universitat Jaume I, Castelló, Spain; 3 Institut de Biomecànica de València, València, Spain; Virginia Tech, UNITED STATES

## Abstract

The taxonomy of foot shapes or other parts of the body is important, especially for design purposes. We propose a methodology based on archetypoid analysis (ADA) that overcomes the weaknesses of previous methodologies used to establish typologies. ADA is an objective, data-driven methodology that seeks extreme patterns, the archetypal profiles in the data. ADA also explains the data as percentages of the archetypal patterns, which makes this technique understandable and accessible even for non-experts. Clustering techniques are usually considered for establishing taxonomies, but we will show that finding the purest or most extreme patterns is more appropriate than using the central points returned by clustering techniques. We apply the methodology to an anthropometric database of 775 3D right foot scans representing the Spanish adult female and male population for footwear design. Each foot is described by a 5626 × 3 configuration matrix of landmarks. No multivariate features are used for establishing the taxonomy, but all the information gathered from the 3D scanning is employed. We use ADA for shapes described by landmarks. Women’s and men’s feet are analyzed separately. We have analyzed 3 archetypal feet for both men and women. These archetypal feet could not have been recovered using multivariate techniques.

## 1 Introduction

A fundamental issue in the appropriate design of footwear is to know foot shape. In particular, it is important to know the types of foot shapes and how the different feet of users can be explained by this taxonomy, i.e. the foot shape distribution. It is not only important from the shoe manufacturing point of view, since an improper fit prevents shoe purchase, but also because poorly fitting footwear can cause foot pain and deformity [1], especially in women. Therefore, numerous studies have been carried out on foot shapes [2–7].

Identifying foot shapes has a significant impact on design [8–10]. A small group of human models that represents the anthropometric variability of the target population is commonly used in ergonomic design and evaluation. Working with a small group of cases, the test cases, provides designers with an efficient way to develop and evaluate a product design. Considering the boundary cases or the extreme cases is a common strategy in design [11]. The idea behind considering the boundary cases is that if the design fits for the extreme cases well, then all other less extreme body types in the target population should also be well accommodated.

Knowledge of the types of body part shapes is not only important in the design or apparel industry [12, 13], but also in ergonomics in general [14, 15], and other disciplines such as sport [16–18], medicine [19–21], phylogeny [22], criminalistics [23], etc. Face classification is also important due to its application in forensic anthropology, crime prevention and new human-machine interaction systems and online activities like e-commerce, e-learning, gaming, dating and social media [24, 25]. Furthermore, taxonomy is also very important not only in anthropometry, but also in morphometry in general, such as in animal or plant taxonomy [26, 27] or also in genetics [28].

The method of establishing types of feet, or other parts of the body, is usually based on subjective or visual elements [29]. When objective techniques have been contemplated, these have been very simple [30]. In fact, despite performing 3D scans, that information is then summarized into a series of multivariate measures [5, 7, 31]. These measures are then treated in an ad hoc, heuristic way to couple pre-established types [12], or a cluster analysis is applied to these measures directly or after applying factor analysis or principal component analysis (PCA) to reduce the dimension [2, 14, 24, 32–35].

Our aim is to improve on the previous methodologies used to define taxonomies by removing the subjective steps and making the data speak for themselves. We use archetypoid analysis (ADA) for shapes based on landmarks, which was developed by some of the authors in [36]. ADA is a variant of archetype analysis (AA), which is an unsupervised statistical learning tool. Archetypes lie on the boundary of the convex hull of the data, meaning that they are extreme profiles. ADA returns archetypes in data. On the one hand, this statistical tool allows us to consider all the information contained in the 3D scanners, without the need for extracting variables from them, thus avoiding the step of deciding which variables may or may not be relevant. On the other hand, the tool itself will provide the taxonomy from the data themselves, i.e. it will provide the existing archetypes in the data, while the user only intervenes to specify the number of archetypes to consider. If the user is not sure how many archetypes should be considered, the tool can provide the most reasonable number of archetypes based on the elbow criterion, which will be explained below. Furthermore, the technique returns how the feet are formed as a function of the archetypes by using mixtures of archetypes. In other words, each foot will be represented as a percentage of the archetypal feet; in this way, it can be easily understood by any user who is not expert in this technique. Despite the fact that clustering is the usual technique for defining typologies, we will use a toy example to show that AA or ADA, rather than cluster analysis (CLA), is the most appropriate statistical technique for obtaining a taxonomy. We will use ADA instead of AA in our problem with 3D scans, because we prefer to obtain archetypal feet corresponding to particular individuals in order to describe those archetypal feet by some multivariate measures a posteriori.

The objective of ADA is to represent the cases by means of a convex combination (a mixture) of archetypes that are actual cases, which are referred to as archetypoids. This makes the results returned by ADA easily interpretable, even for non-experts. The difference between AA and ADA is that in AA the archetypes are mixtures of cases, and therefore, they are not necessarily actual cases. In other words, ADA represents the data as mixtures of extreme cases, and not as mixtures of mixtures, as AA does. AA was defined for multivariate data by [37], while ADA was proposed by [13]. ADA has been extended to other kind of data, such as functions [38] or shapes defined by landmarks [36].

AA and ADA applications have been growing at a great rate and they can be found in a diverse range of disciplines, such as biology [39], computer vision [40–45], developmental psychology [46], engineering [11, 13, 47, 48], finance [49], genetics [50], global development [51], machine learning problems [52], market research [53], multi-document summarization [54], neuroscience [55, 56] and sports [57–59].

Archetypal analysis techniques lie somewhere in between two well-known unsupervised statistical techniques: PCA and CLA. Data decomposition techniques aim to find the latent components, and data are expressed as a linear combination of several factors. The constraints on the factors and how they are combined determine the definition of different statistical techniques. In PCA, factors are linear combinations of variables, and therefore their restrictions are minimal. This compromises the interpretability of the factors, but it helps explain the variability of the data. Instead, in CLA, such as *k*-means algorithm, factors have the greatest restrictions. As factors in *k*-means are centroids (means of groups of data), they are easily interpretable. However, the modeling flexibility of CLA is reduced due to the binary assignment of data to the clusters. In contrast, AA and ADA enjoy higher modeling flexibility than CLA but without losing the interpretability of their factors. [52] and [13] provide a table summarizing the relationship between several unsupervised multivariate techniques. ADA is also compared with many other unsupervised multivariate techniques in [13].

Percentiles should not be used to find the boundary cases in design since with the exception of 50*th*-percentiles, percentile values are not additive [60–62]. Although, different alternatives have been considered, such as the use of CLA [63], the most common approach is based on the use of PCA [61, 64–68]. In this approach, several extreme points are selected from the projection into the first principal components. However, the PCA-approach has several drawbacks [69]. In [61, 67, 68] only the variation in the first two or three components is taken into account, so unconsidered variation may represent cases that are difficult to accommodate, which would be missing. In addition, the number of selected boundary cases with two PCs is eight (fourteen with three PCs), which could be too high in practice. A large numbers of test cases may overwhelm the designer and thus be counterproductive. With ADA we will obtain the extreme cases, since this is precisely the objective of this statistical technique, and we can control the number of extreme cases that the designer wants to consider.

### Toy example

**Fig 1:**
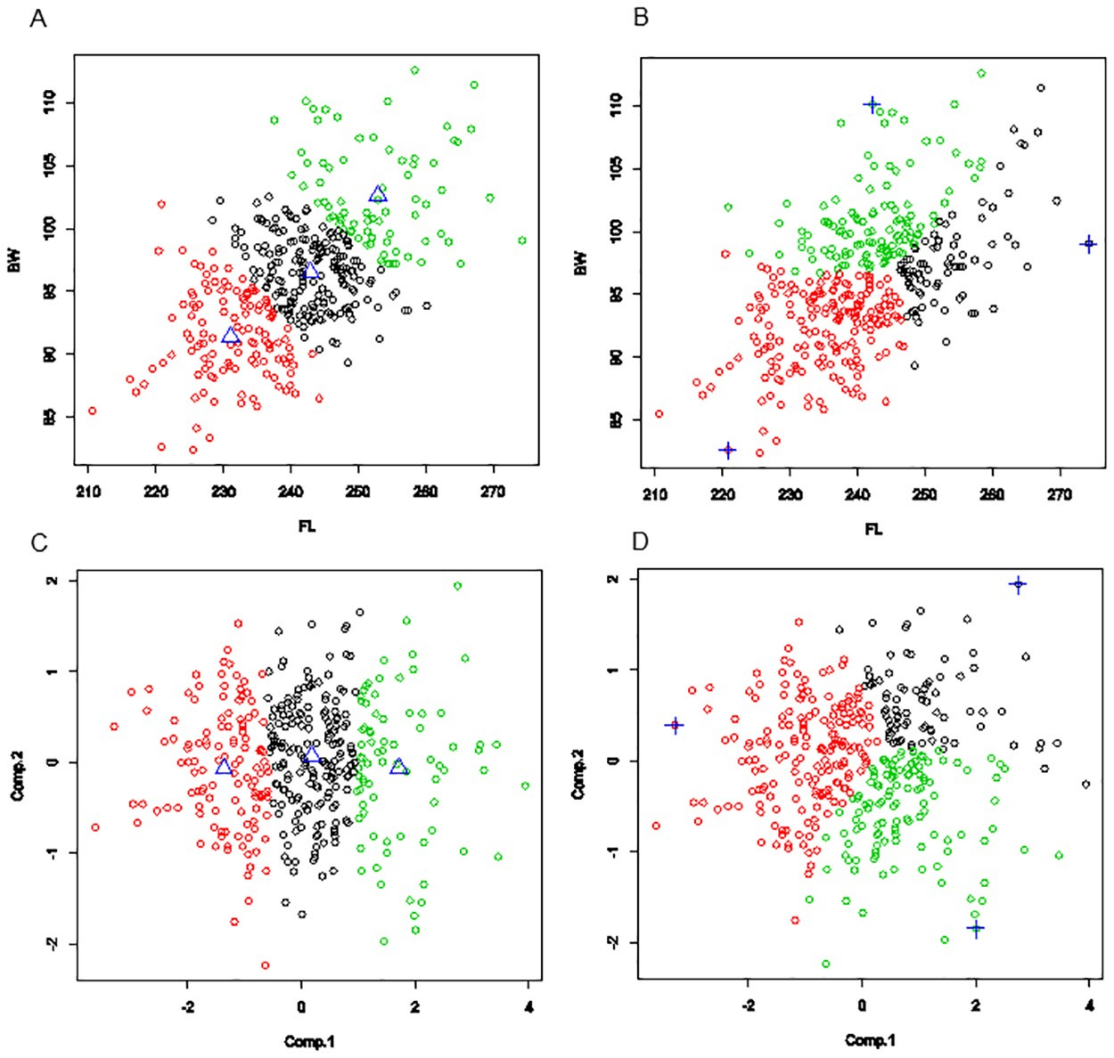
Toy example. (A) Plot of the *k*-means cluster assignments. The blue triangles represent the centroids of each cluster. (B) ADA assignments by the maximum alpha (see Section 2), i.e. assigned to the archetypoid that best explains the corresponding observation. The blue crosses identify the archetypoids. (C) PC scores with cluster assignments. Projected centroids are represented by blue triangles. (D) PC scores with the ADA assignments. Projected archetypoids are represented by blue crosses.

In Fig 1 a toy two-dimensional data set is used to illustrate what archetypoids mean and the differences compared with PCA and CLA, as well as to provide some intuition on what these pure and extreme patterns imply in Anthropometry. Two numeric variables are considered from the data set described below: the Foot Length (FL) and Ball Width (BW) of 382 right feet from the adult female Spanish population. We apply *k*-means and ADA with *k* = 3, i.e. we find 3 clusters and archetypoids, with standardized data. We also apply PCA.

Archetypoids are feet with extreme values, which have clear profiles: archetypoid 1 is characterized by very low FL and BW values, archetypoid 2 has a very high value for BW, but a medium value for FL, while the third archetypoid has a very high FL value together with a medium-high value for BW. Archetypoids are the purest feet. The rest of the feet are expressed as mixtures (collected in alpha coefficients, which is explained in Section 2) of these ideal feet. For example, a foot with values of 244.2 and 86.5 for FL and BW, respectively, is explained by 43% of archetypoid 1 plus 57% of archetypoid 3. From the clustering point of view this foot is assigned to cluster 1, although it is near the border of cluster 2, but clustering does not say anything about the distance of this point with respect to the assigned centroid, or in which direction they are separated. In fact, that foot is quite far from its assigned centroid. This happens because the objective of clustering is to assign the data to groups, not to explain the structure of the data more qualitatively.

**Fig 2:**
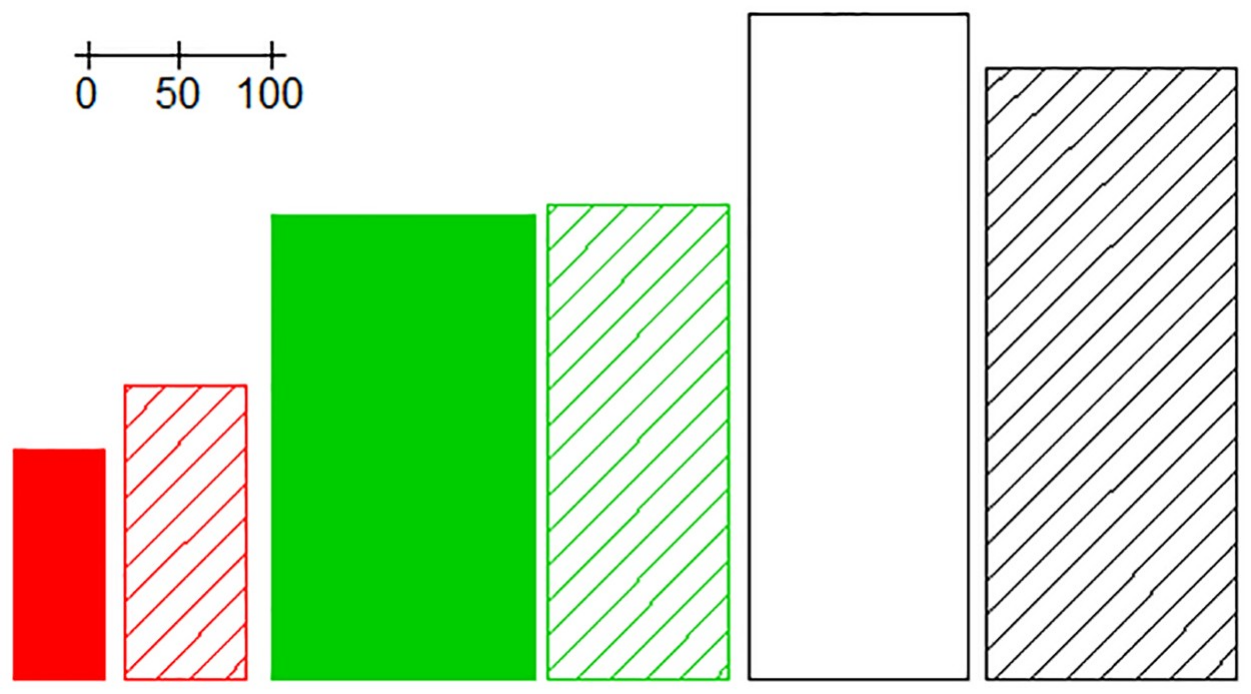
Representative feet of the toy example. The color code of each representant coincides with the color code used in the assignments of Fig 1. The centroids of each cluster are represented by shading lines, while the archetypoids are represented by solid colors. In order to highlight the differences and make them more easily perceptible to the human eye, the percentiles of each representative foot were computed. The rectangles represent the increase or decrease with respect to the median foot measurements, which are: 96 mm (BW) and 241 mm (FL). For example, the percentiles of the first archetypoid are 1 and 2 for each variable, respectively. Therefore, in the plot, it is represented as: 96 ⋅ (1 + (0.01-0.5)) = 49 and 241 ⋅ (1 + (0.02-0.5)) = 125.

This is compatible with the natural tendency of humans to represent a group of items by its extreme units [70]. Fig 1B shows the partition of the set generated by assigning the cases to the archetypoid that best explains each observation. However, when we apply *k*-means to this kind of data set, without differentiated clusters, the centroids are in the middle of the data cloud. Centroid profiles are not as differentiated from each other as archetypoid profiles. This happens because centroids have to cover the set in such a way that the set is partitioned by minimizing the distance with respect to the assigned centroid (see [71] about the connection between set partitioning and clustering). On the one hand, this means that the set partition generated by *k*-means and ADA would be different (Fig 1A and 1B). On the other hand, centroids are not the purest, and therefore their profiles are not as clear as those of archetypoids. In Fig 2 we show the foot centroids and archetypoids as rectangles. Archetypoids are more intuitively interpretable due to the extremeness of their dimensions: the first archetypoid is a very short and narrow foot (smaller in both dimensions than the smallest centroid); the second archetypoid is very wide, while the second centroid is similar to the mean foot; and the third archetypoid is a very long foot that is longer than the third centroid. All the foot centroids have the same aspect, i.e. the same FL and BW ratio as the mean foot. However, this is not the case with ADA. Archetypoid 1 has the same ratio as the mean foot, but not archetypoids 2 and 3, which are more flattened and elongated, respectively. This can be clearly appreciated in the PC projections of Fig 1C and 1D. The first PC is a size component composed of the addition of FL and BW (the loadings are 0.7 and 0.7), while the second PC is a shape component composed of the contraposition of FL and BW (the loadings are 0.7 and -0.7). Note that centroids are all in the zero horizontal line, i.e. centroids do not account for different shapes. However, archetypoids are distributed on the border of the PC score space. Archetypoid 1 is on the zero horizontal line, but with a lower score in PC 1 than the centroids. Archetypoids 2 and 3 have higher scores in PC 1 than the centroids, and additionally they have no zero scores in PC 2, being negative for archetypoid 2 and positive for archetypoid 3. Note also that the feet projected on the first quadrant of the PC space correspond to feet similar to archetypoid 3, those projected on the fourth quadrant correspond to feet similar to archetypoid 2, while the second and third quadrant of the PC space correspond to feet similar to archetypoid 1. The mean foot, located at the origin, coincides with the intersection where the three partitions meet them, i.e. the mean foot is a balanced mixture between the three archetypoids. Finally, note that archetypoids do not coincide with the individuals with the most extreme PC scores (see Fig 1D). Unlike PCA, the objective of ADA is to obtain extreme cases, and individuals with extreme PCA scores do not necessarily return archetypical observations. In fact, archetypes could not be recovered with PCA even if all the components had been considered [11]. Therefore, the appropriate statistical technique for obtaining the extreme cases is ADA.

The outline of the paper is as follows: In Section 2 we introduce our data and review ADA for real-valued multivariate data and for shapes defined by landmarks. In Section 3, our proposal is applied to our women and men data sets from the 3D foot scanner and the results are discussed. Section 4 contains conclusions and some ideas for future work.

## 2 Materials and methods

### Foot database

**Fig 3:**
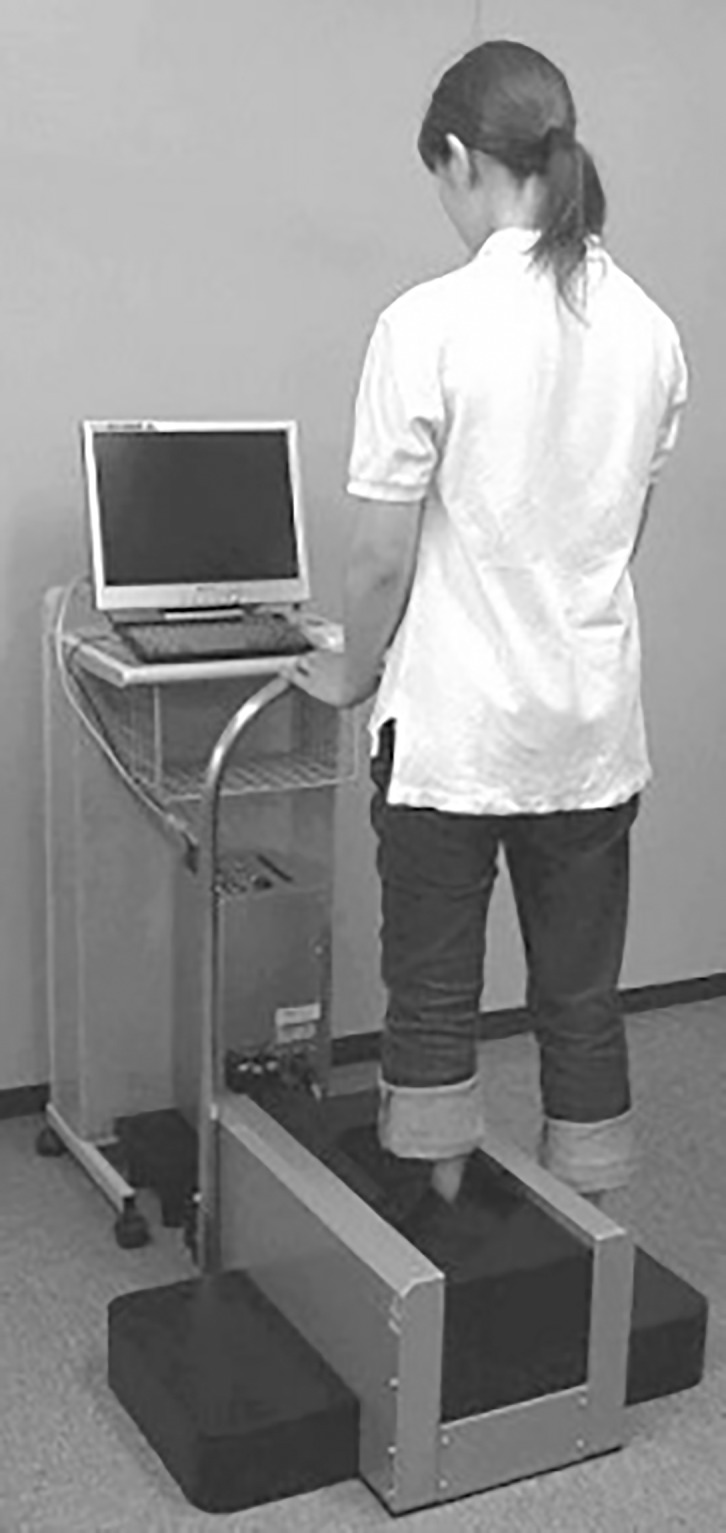
Infoot^®^ scanner. Scanner used to obtain the foot scans.

**Fig 4:**
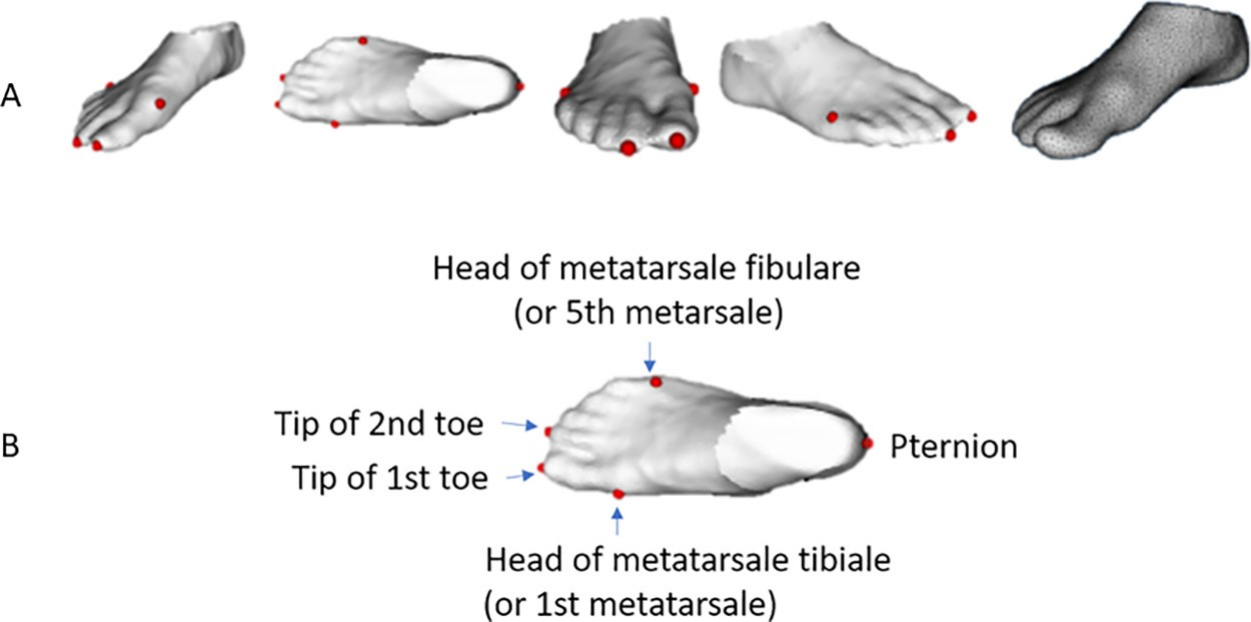
Foot landmarks. (A) Foot landmarks used in the registration of the database and foot template topology (the last image). (B) Names of the five foot landmarks.

Our anthropometric database is composed of 775 3D right foot scans representing the Spanish adult male and female population, 393 corresponding to men and 382 to women. The mean, standard deviation, minimum and maximum age for women (men) were: 40.8 (42.3), 11.3 (10.1), 19 (19) and 68 (67), respectively. The data set was collected from May 3rd 2006 to July 21st 2006 by IBV in the project ‘Estudio antropométrico y morfológico 3D de los pies de la población española para su aplicación al diseño de calzado y componentes’ (IMPRDA/2005/38) funded by Valencia Region Government (i.e. Instituto de la Mediana y Pequeña Industria Valenciana, IMPIVA) under the programme ‘Ayudas a la Promoción del Diseño en la Comunidad Valenciana’. All participants signed an informed consent complying with existing Spanish legislation (Ley Orgánica 15/1999, de 13 de diciembre, de Protección de Datos de Carácter Personal, LOPD) granting the use of the data for research purposes. The data were collected by IBV from volunteers recruited in different regions across Spain at shoe shops and workplaces using an INFOOT laser scanner [72]. The scanning process is carried out as can be seen in Fig 3: the user stands upright placing equal weight on each foot, in a specific position and orientation. We obtain a 3D point cloud representing the complete outer surface of the foot, including the sole of the foot. Prior to foot scanning, an expert placed five landmarks at key anatomical locations: tip of the first toe, tip of the second toe, head of the metatarsale tibiale, head of the metatarsale fibulare and pternion (see Fig 4). The landmarks used were non-reflective stickers with a 5 mm diameter provided by the distributor of the 3D foot scanner [72]. The spatial location of theses landmarks was automatically detected and recorded by the software of the 3D scanner. The accuracy of anatomical landmark location in human feet by experts is complex to assess. While [73] reported a median intra-observer error of 2-3 mm, we estimate that our expert had an accuracy of at least 5mm. No personal data was gathered along with the 3D point cloud.

**Fig 5:**
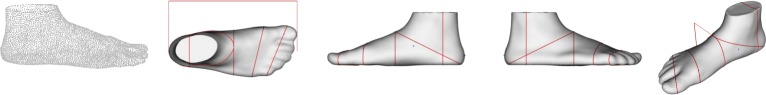
Foot measurements. Examples of digital measurements elicited from a 3D registered foot. Only 8 of the 22 measurements will be used in Section 3, where they will be described in detail. These 8 measurements correspond to the variables that could most influence shoe fitting according to shoe design experts.

3D foot shapes were registered using the method reported by [74] with a template made up of 5626 vertices, using the five foot landmarks, which enables the automatic computation of 22 key foot measurements (see Fig 5). Put simply, we register the original unorganized point clouds to a common template (template fitting process), which is initialized and guided by the five anatomical landmarks. The template mesh was obtained by uniformly remeshing a watertight mesh representing one foot of the sample. A foot that was randomly selected among those that had an average length and that did not present mild foot conditions such as bunions, hammer toes, claw toes, cavus foot or flat foot. This method provides sufficient template fitting accuracy. The mean, root-mean-square and maximum Hausdorff distance from the scanned point cloud to the registered template are approximately 0.07, 0.1 and 1 mm, respectively, which provides sufficient template fitting accuracy for objects scanned with a resolution of 0.5-1 mm.

The 22 foot measurements are used in product design and in clinical assessment. All 3D registered feet were digitally measured with the algorithms developed by the IBV (Biomechanics Institute of Valencia). Unlike body measurements, foot measurements are not standardized. Only Foot Length, Ball Girth and Ball Width are considered in [75], [76] and [77]. The definitions are those used by the Human Shape Lab of the IBV, which comply with standards and are compatible with the accepted definitions found in the literature [78–82].

However, in contrast to the common procedure in the literature, our working data are not the multivariate measurements, which are a mere summary of the richer information contained in the 3D foot scans. Our data set are the set of landmarks; the foot shape of each individual in our data set was represented by 5626 3D landmarks, i.e. by a 5626 × 3 configuration matrix. Therefore, we work with 775 configuration matrices.

Other researchers can obtain the data set in the same way. The data set is saved as an R object (.Rdata) [83], in a matrix where each row corresponds with each individual and variables are in columns. The data sets and code in free and open software R [83] for reproducing the results are available at https://figshare.com/articles/adafeet_rar/11553324. Note that the availability of the code that implements the methodology allows the methodology to be applied to any data set. In order to demonstrate the procedure in the code we carried out a systematic sample of the landmarks and we retained 5% of the landmarks, since the same results, archetypoids, are obtained using 5626 landmarks and 282 landmarks. In this way, if anybody wants to reproduce the results, they can obtain the solution faster. Raw data obtained through project IMPRDA/2005/38 are available on request at ibv@ibv.org.

### ADA in the shape space

In the multivariate context, let ${\left\{x_{i}\right\}}_{i=1}^{n}$ be a set of observations of a variable vector in $R^{k}$ taken on *n* individuals, that is, each observation consists of *k* measurements **x**_*i*_ = (*x*_*i*1_, *x*_*i*2_, …, *x*_*ik*_). The archetypoids, {**z**_*j*_}_*j*=1,⋯,*p*_, are observed data points, so that observations can be approximated by convex combinations of the archetypoids. Then, we will define two matrices of coefficients *β* and *α*, such that $x_{i}\approx \sum_{j=1}^{p}\alpha_{ij}z_{j}$ and $z_{j}=\sum_{l=1}^{n}\beta_{jl}x_{l}$, with *β*_*jl*_ ∈ {0, 1}, ∀*j*, *l*. To estimate both matrices of coefficients, the following mixed-integer minimization problem of the residual sum squares (RSS) has to be solved:

$$\begin{array}{c} RSS=\sum_{i=1}^{n}\|x_{i}-\sum_{j=1}^{p}\alpha_{ij}z_{j}{\|}^{2}=\sum_{i=1}^{n}{\left\|x_{i}-\sum_{j=1}^{p}\alpha_{ij}\sum_{l=1}^{n}\beta_{jl}x_{l}\right\|}^{2}, \end{array}$$ (1)

under the constraints

1. $\sum_{j=1}^{p}\alpha_{ij}=1$ with *α*_*ij*_ ≥ 0 and *i* = 1, …, *n* and
2. $\sum_{l=1}^{n}\beta_{jl}=1$ with *β*_*jl*_ ∈ {0, 1} and *j* = 1, …, *p*.

Note that *β*_*jl*_ = 1 for one and only one *l*, otherwise *β*_*jl*_ = 0.

However, as stated above, our data are not multivariate measurements, but a set of landmarks.

**Fig 6:**
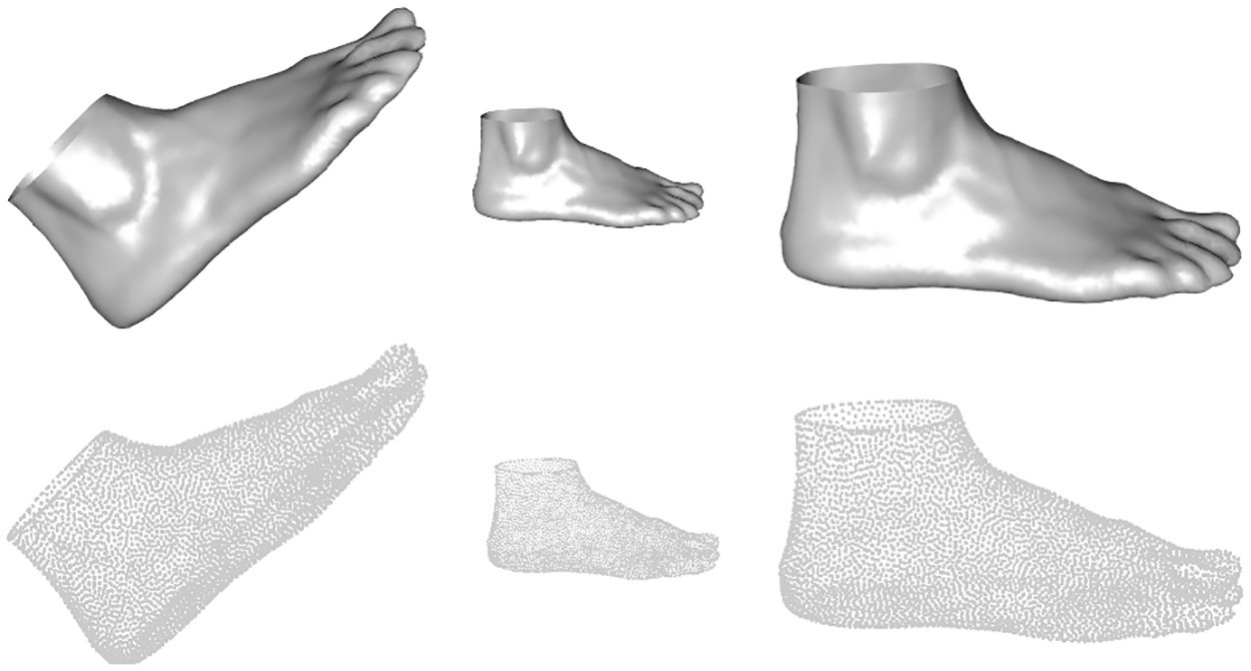
Three feet with the same shape. All the objects in this figure correspond to the same shape, i.e. they are equivalent; however, their 3D coordinates are different.

Let **X**_1_, …, **X**_*n*_ be *n* = 775 *k* × 3 configuration matrices, each matrix containing the 3D coordinates of the *k* = 5626 landmarks of each foot. Each matrix could be rearranged to convert it into a vector in $R^{3k}$ and the above definitions of archetypoids could be used. Nevertheless, these matrices are not representative of the shape of the feet because any translation, rotation or rescaling of them has the same shape. An example can be seen in Fig 6.

Hence, from a theoretical point of view we can define the shape space as:

**Definition 1** *The shape space* $\Sigma_{3}^{k}$ *is the set of equivalence classes* [*X*] *of k* × 3 *configuration matrices* $X\in R^{3\times k}$ *under the action of Euclidean similarity transformations (translation, rotation and scale change)*.

In order to obtain a representative element of the shape [*X*] of a foot, all these transformations have to be removed.

First we remove the location effect. There are different ways to remove location, but we will use the most convenient for mathematical reasons, consisting of multiplying the configuration matrix by the (*k* − 1) × *k* Helmert sub-matrix [84], *H*, i.e. *X*_*H*_ = *HX*. After removing the location, the representative of a foot is now a 3 × (*k* − 1) matrix that could be regarded as a vector in the Euclidean space $R^{3(k-1)}$.

To filter scale we can divide *X*_*H*_ by its Frobenius matrix norm, which is the centroid size, *S*(*X*) = ‖*X*_*H*_‖:

$$\begin{array}{c} Y=\frac{X_{H}}{\|X_{H}\|}. \end{array}$$ (2)

*Y* is called the pre-shape of the configuration matrix *X* because all information about location and scale is removed, but rotation information remains.

It is important to note that when scale is removed, the representative of the shape of the foot is still a (*k* − 1) × 3 matrix, but it cannot be regarded as a vector in a Euclidean space. We are restricted to matrices with the Frobenius norm equal to one and, as a result, they are points in the hypersphere *S*^3(*k*−1)^ of $R^{3(k-1)}$ (a curved subspace). Mathematically, a sphere is a Riemannian manifold.

To choose a single representative of [*X*] we need to eliminate the rotations and, as a result, our data would be points on the quotient space *S*^3(*k*−1)^/*SO*(3) where *SO*(3) is the special orthogonal group of rotation matrices.

**Fig 7:**
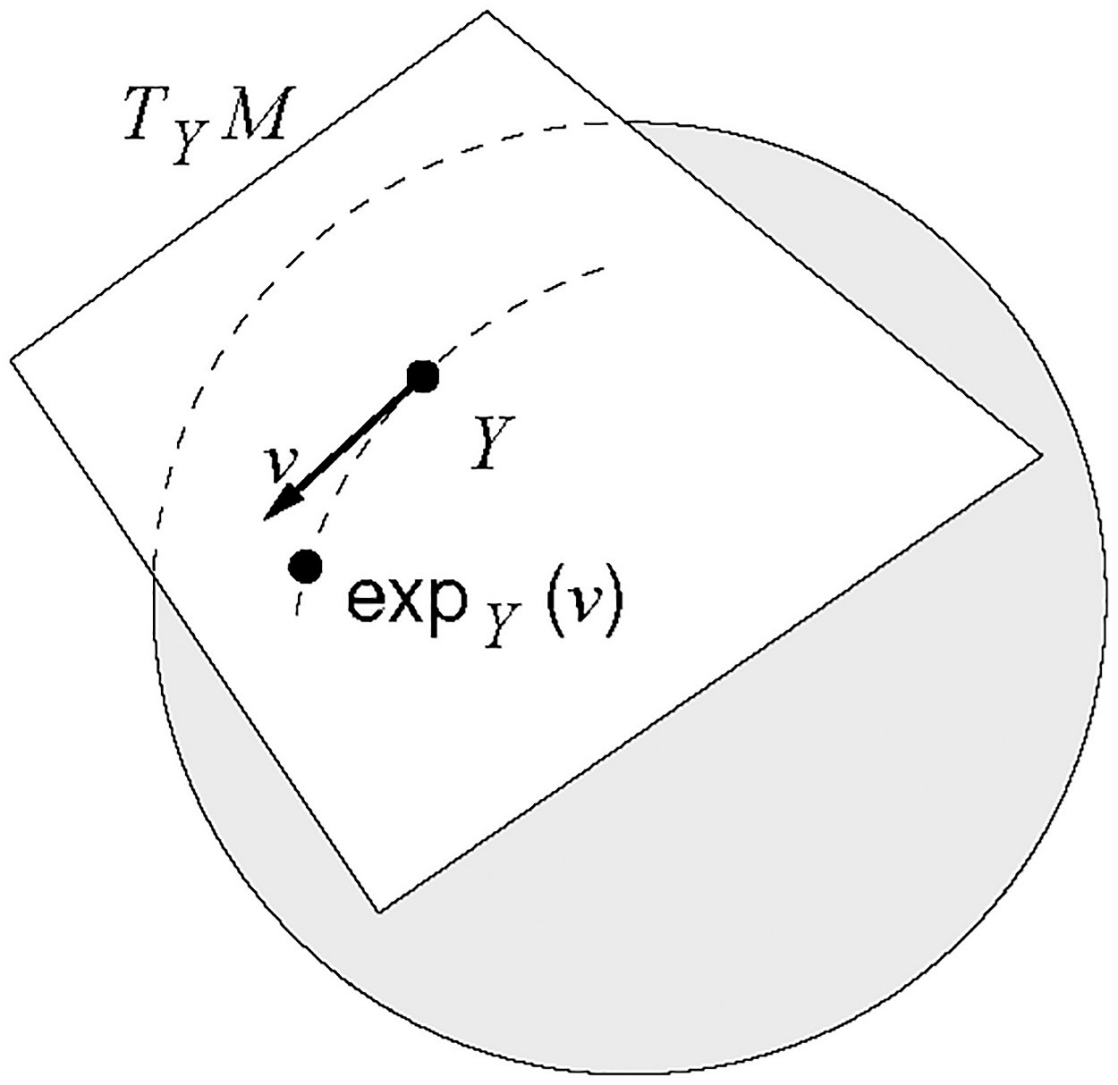
Tangent space at point *Y* on a sphere. A geometrical view of the tangent plane to a Riemannian manifold *M* (*S*^3(*k*−1)^ in our case) at a point *Y*, together with the exponential map.

Mathematically, this space is a Riemannian submersion of the sphere. The curvature of this space makes the data behave differently than they would do in the Euclidean space; for example, neither the sum nor the multiplication by a scalar is defined i.e. the shape space is not a vectorial space. Fortunately, the theory of Riemannian manifolds tells us that it is possible to work locally in a Riemannian manifold as if we were in a Euclidean space, using the projections of the tangent space at a given point. See Fig 7.

The full Procrustes mean in *S*^3(*k*−1)^/*SO*(3) of a set of configuration matrices *X*_1_, …, *X*_*n*_ can be defined by

$$\begin{array}{c} \left[\hat{\mu }\right]=\arg\underset{\mu :S(\mu )=1}{\inf}\sum_{i=1}^{n}d_{F}^{2}\left(X_{i},\mu \right), \end{array}$$ (3)

where *d*_*F*_ stands for the full Procrustes distance. The mean is estimated by an iterative procedure as described by [85] on pp.90-91. The full Procrustes distance between two configuration matrices *X*_1_ and *X*_2_ is defined by:

$$\begin{array}{c} d_{F}\left(X_{1},X_{2}\right)=\underset{R\in SO(3),\beta \in R}{\inf}\left\|Y_{2}-\beta Y_{1}R\right\|, \end{array}$$ (4)

where *SO*(3) is the orthogonal group of rotations. As explained by [85] on pp. 61-62,

$$\begin{array}{c} d_{F}\left(X_{1},X_{2}\right)=\sqrt{1-{\left(\sum_{i=1}^{m}\lambda_{i}\right)}^{2}}, \end{array}$$

where λ_1_ ≥ λ_2_ ≥ … λ_*m*−1_ ≥|λ_*m*_| are the square roots of the eigenvalues of $Y_{1}^{T}Y_{2}Y_{2}^{T}Y_{1}$, and the smallest value λ_*m*_ is the negative square root if and only if $Y_{1}^{T}Y_{2}<0$.

So, in view all the above, in [36] we introduced ADA in the tangent space on the mean shape, assuming that our data are sufficiently concentrated around the mean to consider the tangent space a good approximation to shape space. Let us review the main points of this result.

The map that allows us to move from the tangent space to the manifold is called the *exponential map*. And the inverse of the exponential map is called the *logarithmic map*. Their expressions for the shape space are given below.

Let *S* be the pre-shape of the Procrustes mean *μ* and *Y*_1_, …, *Y*_*n*_ the preshapes of *X*_1_, …, *X*_*n*_, obtained using Eq 2. To obtain the expression of the projection onto the tangent plane at *S* of *X*_1_, …, *X*_*n*_, the pre-shape *Y*_*i*_ is rotated to be as close as possible to *S*.

We write the rotated pre-shape as $Y_{i}\hat{\Gamma_{i}}$. The expression of $\hat{\Gamma_{i}}$ can be found on p. 61 of [85]:

$$\begin{array}{c} \hat{\Gamma_{i}}=U_{i}V_{i}^{T}, \end{array}$$

where *U*_*i*_, *V*_*i*_ ∈ *SO*(3) are the left and right matrices of the singular value decomposition of *S*^*T*^ *Y*_*i*_.

Then, the Kent’s partial tangent coordinates of *Y*_*i*_ on the tangent space at *S*, **v**_*i*_, which will be used in our work, are:

$$\begin{array}{c} v_{i}=\log_{S}\left(Y_{i}\right)\frac{\text{sin}(trace\left(S^{T}Y_{i}\hat{\Gamma_{i}}\right))}{trace(S^{T}Y_{i}\hat{\Gamma_{i}})}, \end{array}$$ (5)

where log_*S*_(*Y*_*i*_) is defined by:

$$\begin{array}{c} \log_{S}\left(Y_{i}\right)=\left(I_{km-m}-\text{vec}\left(S\right)\text{vec}{\left(S\right)}^{T}\right)\text{vec}\left(Y_{i}\hat{\Gamma_{i}}\right)\frac{trace(S^{T}Y_{i}\hat{\Gamma_{i}})}{\sin(trace\left(S^{T}Y_{i}\hat{\Gamma_{i}}\right))}, \end{array}$$ (6)

where *I*_*km*−*m*_ is the (*km* − *m*) × (*km* − *m*) identity matrix and *vec* stands for the vectorizing operator. The vectorizing operator of an *l* × *m* matrix *A* with columns *a*_1_, *a*_2_, …, *a*_*m*_ is defined as: $\text{vec}\left(A\right)={\left(a_{1}^{T},a_{2}^{T},\ldots ,a_{m}^{T}\right)}^{T}$.

To project back a point v in the tangent space to the shape space, the exponential map must be used:

$$\begin{array}{c} Y_{v}={\text{vec}}^{-1}\left({\left(1-v^{T}v\right)}^{1/2}\text{vec}\left(S\right)+v\right). \end{array}$$ (7)

Finally, the configuration matrix representing **v** would be:

$$\begin{array}{c} X_{v}=H^{T}Y_{v}. \end{array}$$ (8)

Let **v**_1_, …, **v**_*n*_ be the tangent coordinates of **X**_1_, …, **X**_*n*_. The coordinates in the tangent space **u**_*j*_ *j* = 1, ‥, *p* of the archetypoids $Z_{j}\in \Sigma_{3}^{k}$, *j* = 1, ‥, *p* are obtained by minimizing:

$$\begin{array}{c} RSS=\sum_{i=1}^{n}\|v_{i}-\sum_{j=1}^{p}\alpha_{ij}u_{j}{\|}^{2}=\sum_{i=1}^{n}{\left\|v_{i}-\sum_{j=1}^{p}\alpha_{ij}\sum_{l=1}^{n}\beta_{jl}v_{l}\right\|}^{2}, \end{array}$$ (9)

under the constraints

1. $\sum_{j=1}^{p}\alpha_{ij}=1$ with *α*_*ij*_ ≥ 0 and *i* = 1, …, *n* and
2. $\sum_{l=1}^{n}\beta_{jl}=1$ with *β*_*jl*_ ∈ {0, 1} and *j* = 1, …, *p*.

As archetypoids are actual individuals of the sample, the projection of the obtained archetypoids from the tangent space back into the configuration space is immediate.

In summary, we apply multivariate ADA in a tangent space to the shape space.

## 3 Results and discussion

We have applied ADA separately for men and women, since previous studies have shown gender foot shape differences [5, 7]. Furthermore, footwear designers usually propose different designs for women and men. We have analyzed the whole sample as representative of the population, without removing any possible outlier, since this could be considered part of the population variability. If we were more interested in the archetypal feet of the majority than of the totality, outliers could be identified by computing the Procrustes distances of each foot to the mean, as in [36]. In the same way, if we wanted to accommodate a certain percentage of the population, then only an appropriate part of the sample could be used.

**Fig 8:**
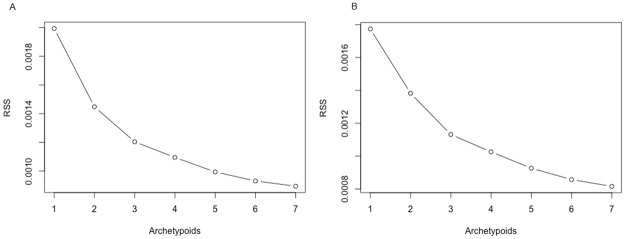
Screeplots for ADA with 3D landmarks. (A) Screeplot for women. (B) Screeplot for men.

In order to determine the number *p* of archetypoids for women and men, RSS values have been represented for a series of different *p* values in Fig 8. Although not very clear, it seems that an elbow is found for *p* = 3, for men and women. In any case, a shoe design expert indicated that this would be a reasonable number for design purposes (a large number of representative cases may overwhelm the designer and thus be counterproductive [11]). Therefore, in the interests of brevity, we examine the results of 3 archetypoids. If the designer decided to choose more archetypoids, our procedure would be the same.

**Fig 9:**
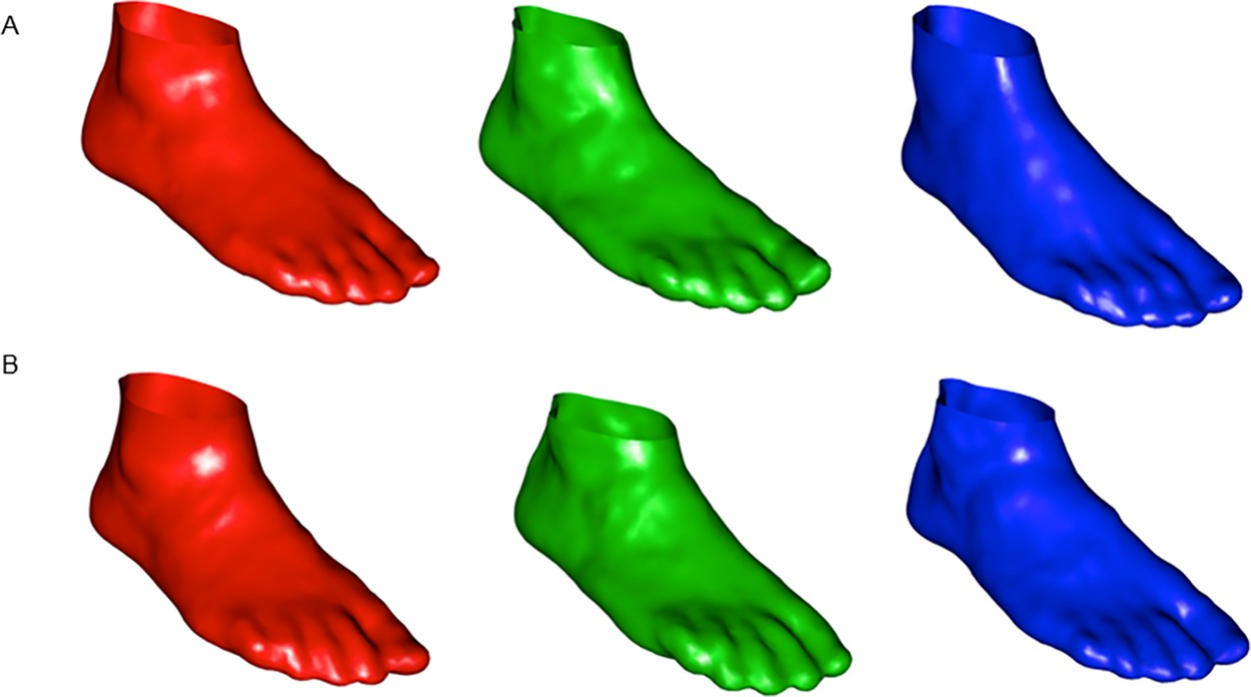
Three archetypoids obtained with 3D landmarks. (A) Archetypoids for women. (B) Archetypoids for men. The first archetypoids are shown in red, the second archetypoids in green, while the third archetypoids are shown in blue.

The three archetypoids for women and men are displayed in Fig 9. Archetypoids correspond to actual individuals, so in order to get a concise description of the archetypoids, rather than the whole set of 22 variables, we have computed the percentiles of the most relevant variables in shoe design. According to shoe design experts, the variables that could most influence shoe fitting are: Foot Length, FL (distance between the rear and foremost point the foot axis); Ball Girth, BG (perimeter of the ball section); Ball Width, BW (maximal distance between the extreme points of the ball section projected onto the ground plane); and Instep Height, IH (maximal height of the instep section, located at 50% of the foot length). But the following variables are also relevant: Toe Height, TH (maximal height of the toe section); Ball Position, BP (distance from the rearmost point of the foot to the intersection of the ball section and the foot axis); Instep Girth, IG (perimeter of the instep section, located at the 50% of foot length); and Instep to Heel Girth, IHG (perimeter of the section that passes through the heel to the instep, located at 50% of the foot length). According to footwear experts, the variable that best describes the size of the foot is FL. As the shape corresponds to the geometrical information that remains once the scale is eliminated, to describe the archetypal foot shapes by variables, we consider the rest of the variables after removing the scale by dividing each of the variables by FL: BG/FL, BP/FL, BW/FL, IG/FL, IH/FL, IHG/FL and TH/FL. Table 1 shows the percentiles of the 3 archetypoids for those variables for women and men, respectively.

**Table 1:** Percentiles corresponding to the 3 archetypal foot shapes of women and men obtained using 3D landmarks.

Archetypoids	BG/FL	BP/FL	BW/FL	IG/FL	IH/FL	IHG/FL	TH/FL
A1W	24	13	32	36	32	62	58
A2W	74	9	83	42	11	7	45
A3W	26	99	45	28	3	7	6
A1M	22	15	31	38	28	67	58
A2M	75	10	86	44	10	5	46
A3M	25	98	45	29	4	5	3

The first three archetypoids correspond to the sample of women. They are denoted by A1W, A2W and A3W. The last three archetypoids correspond to the sample of men. They are denoted by A1M, A2M and A3M. See details in the text about the meaning of the variables.

According to the percentile profiles (the percentiles of A1W and A1M, A2W and A2M, and A3W and A3M are very much alike), the three archetypoids found for men and women are quite similar. This could indicate that in global terms the three extreme foot shapes for men and women resemble each other. For a larger *p* values the majority of profiles coincide for men and women but some are different, showing different shapes between genders. Nevertheless, as stated before, we concentrate on the results for *p* = 3 for footwear design in order to create a design that could fit the three archetypal feet.

The percentile profile of the first archetypoid for both women and men is characterized by medium-low percentiles for variables BG/FL, BW/FL, IG/FL, IH/FL, medium-high percentiles for variables IHG/FL and TH/FL, and a low percentile for BP/FL. The percentile profile of the second archetypoid for both women and men is characterized by high percentiles for BG/FL and BW/FL, very low percentiles for BP/FL, IH/FL and IHG/FL and, medium percentiles for IG/FL and TH/FL. Finally, the percentile profile of the third archetypoid for both women and men is characterized by low percentiles for variables BG/FL and IG/FL, a very high percentile for BP/FL, a medium percentile for BW/FL and, very low percentiles for IH/FL, IHG/FL and TH/FL.

**Fig 10:**
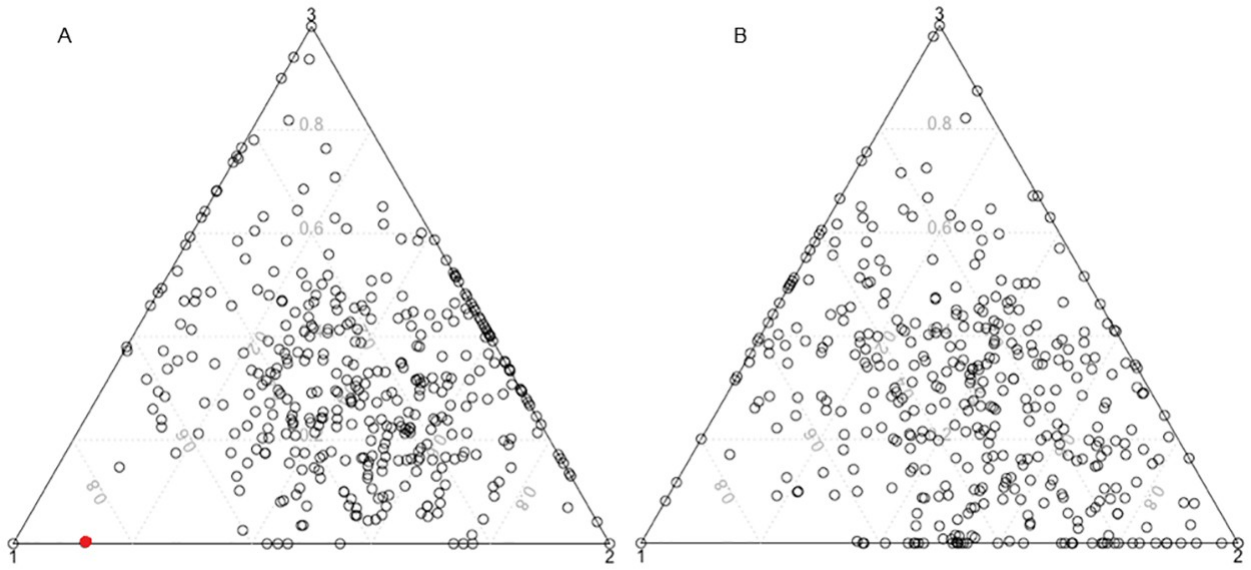
Ternary plots for ADA with 3D landmarks and *p* = 3. (A) Ternary plot for women. (B) Ternary plot for men. Each point corresponds to a foot, which is described by the alpha values. The corners of the triangle indicate the location of each of the archetypoids. For example, in the first ternary plot the red point represents a foot that is approximated by 88% of archetypoid 1 and 12% of archetypoid 2.

In order to view the composition of feet according to the archetypal feet, i.e. to see their distribution, Fig 10 shows the ternary plot for women and men, respectively. The ternary plot represents the alpha values, the sum of which is one, in an equilateral triangle. In both cases, the distributions are quite similar: the majority of feet are a mixture between the three archetypoids, but the second archetypoid has a larger weight than the other archetypoids. There is a small gender difference in the distribution of the purest feet: in women there is a small concentration of feet that are a mixture between archetypoids 2 and 3 (they appear on the side of the triangle that joins archetypoids 2 and 3), but in men this concentration appears on the side of the triangle that joins archetypoids 1 and 2.

Multivariate ADA has been applied to the variables BG/FL, BP/FL, BW/FL, IG/FL, IH/FL, IHG/FL and TH/FL to check if the same results could have been obtained using the variables directly instead of the 3D landmarks. Table 2 shows the percentiles of the 3 multivariate archetypoids for women and men, respectively. The archetypal profiles for men and women coincide again. However, the profiles obtained by multivariate variables and 3D landmarks are somewhat different. The largest differences are found between the profiles of the first archetypoids obtained with multivariate data and 3D landmarks. These differences are found in variables BG/FL, BW/FL, IG/FL, IH/FL, IHG/FL and TH/FL, especially in first four of these variables. The second profiles are similar, with no large differences in variables BG/FL, BW/FL and TH/FL. The third archetypoid profile with 3D landmarks is similar to the third profile obtained with multivariate variables with some not too large differences in variables BG/FL, BW/FL, IG/FL and IH/FL. Therefore, the archetypal profiles obtained using the richer information of 3D landmarks cannot be recovered entirely using multivariate data.

**Table 2:** Percentiles corresponding to the 3 archetypal foot shapes of women and men obtained using variables.

Archetypoids	BG/FL	BP/FL	BW/FL	IG/FL	IH/FL	IHG/FL	TH/FL
A1W	99	18	99	98	99	100	97
A2W	15	9	18	34	5	1	4
A3W	1	100	4	0	32	1	18
A1M	98	36	95	98	97	96	94
A2M	49	2	59	26	4	16	28
A3M	0	99	0	2	26	13	4

The first three archetypoids correspond to the sample of women. They are denoted by A1W, A2W and A3W. The last three archetypoids correspond to the sample of men. They are denoted by A1M, A2M and A3M. See details in the text about the meaning of the variables.

## 4 Conclusions

We have introduced ADA for the taxonomy of foot shapes defined by 3D landmarks. This procedure avoids the subjective steps of previous methodologies, such as the selection of a set of variables from the 3D foot scans. We have shown that ADA is a more appropriate technique for establishing types of feet (or other parts of the body) than the usual clustering techniques.

We have applied ADA to a sample of foot shapes from the Spanish adult population, and we have analyzed the 3 archetypal feet found using 3D landmarks. We have also shown that these archetypal feet could not be recovered using a multivariate technique. Knowing the archetypal feet can help to design adequate footwear to improve fit and accommodate a great percentage of the population.

As future work, the same methodology could be applied to other databases of other parts of the body or to data sets outside the field of Anthropometry. On the other hand, if landmarks are not the only descriptors of the observations, but other information is available, for example color in biological data sets as described by [26] for ladybird beetles, we can extend the methodology and define ADA in this new space. In that case, the objective function in Eq 1 should be modified to take into account both sets of characteristics. Once the shapes are represented in the tangent space, the information of both vectorial spaces could be (weighted) combined using an adequate interior product to build the corresponding RSS.

If we do not have landmarks to describe the shapes, but instead sets or contour functions, archetypal analysis could also be applied. Preliminary work in two-dimensional sets has been carried out in [86] and [87], respectively, but these ideas could be extended to 3D sets or surfaces.
